# Identities of the incumbent and the successor in the family business succession: Review and prospects

**DOI:** 10.3389/fpsyg.2023.1062829

**Published:** 2023-03-17

**Authors:** Weining Li, Yunqiao Wang, Liebing Cao

**Affiliations:** School of Business Administration, South China University of Technology, Guangzhou, Guangdong, China

**Keywords:** family business succession, incumbent, successor, identity perception, SIT, RIT, framework

## Abstract

**Introduction:**

The cognition and motivation of family business incumbents and the successors will directly influence succession behavior, yet they face identity challenges during succession due to the intersection of family and firm context, and their ability to overcome identity challenges will determine the success of the succession. However, as studies on their identity are fragmented and lack systematicity, there is a need to assess the relevant literature.

**Methods:**

Drawing from social identity theory (SIT) and role identity theory (RIT), this article adopts a systematic literature review approach to analyze 99 SSCI-indexed articles to explore family business succession from an identity perspective.

**Results:**

The article finds that the focus on the self-concept of the incumbent and the successor shifts from group identification to role identity perception and multi-roles, and succession behaviors are based on identity perception.

**Discussion:**

This article summarizes a knowledge framework of the antecedents, connotations, and behavioral consequences of identity perception, revealing that family business succession from an identity perspective exhibits psychological and multidisciplinary characteristics, highlighting iterative and mutual features. Based on identity theories and succession research, this article proposes future directions from the research topics, research methods, and theoretical perspectives within the existing knowledge framework, such as cross-cultural and diachronic analysis, as well as from the theoretical perspectives of family, personality development, and pedagogy.

## 1. Introduction

Intergenerational succession has been a major challenge to the continuity and enrichment of family business as it determines the future organizational path for the immediate years or even long-term periods (Chua et al., 1999). As the family firm is the integration of family and firm systems, a unique, valuable, and inimitable resource combination mode can be formed and transmitted in long-term investment and altruistic behavior (Gu et al., 2019). However, family involvement can also be destructive, as the overlap of kinship and working relationships complicates intergenerational interactions and further creates emotional conflict and role cognition bias among family members. This conflict often escalates during succession, resulting in a lack of succession intentions (Gagné et al., 2021), unqualified successors (De Massis et al., 2014), and the reluctance of incumbents to delegate power (Lam, 2011; Alterman et al., 2020). It is a growing practical and academic concern to solve the emotion and role conflict caused by the overlap between family and firm identities during family business succession.

The existing literature on family business succession has explored key assets (Daspit et al., 2016) transit from the senior generation (abbreviated as sen-gen) to the junior generation (abbreviated as jun-gen), such as wealth (Carr et al., 2016), social capital (Schell et al., 2018), and knowledge (Wang and Shibing Jiang, 2018). Some studies have also explored the entrepreneurship outcomes, such as entrepreneurial spirit, entrepreneurial competence, and behavior (Capolupo et al., 2022). However, these studies have discussed key elements that influence succession behavior, but neglect in-depth socio-cognitive contexts, especially identification and identity construction, so fail to explain why succession still proceeds hard and arduously after resource transfer is completed. In fact, succession behavior is based on participants' cognition and emotions (Bee and Neubaum, 2014). And in the context of family businesses, the perception of identity is of particular significance, both at the organizational and individual levels (Bettinelli et al., 2022). Succession studies have addressed identity issues and emotions associated with them. For example, Radu-Lefebvre et al. (2021) pointed out that during succession, identity confirmation can better explain entrepreneurs' emotions, cognition, and behavior, as well as the mechanism of intergenerational interaction in family businesses (Hall, 2012). However, the research topics are scattered and lack a comprehensive overview of identity issues on the sen-gen and the jun-gen.

Identity refers to a set of “meanings” that individuals use to distinguish themselves from others and that help answer the question “Who am I?” or “Who are we?” (Stets and Burke, 2000). Through identity work, individuals perceive, select, and create identity, and once individuals acquire specific identities, they will intentionally act in ways consistent with the norms and institutions of the groups to which they believe they belong (Ethier and Deaux, 1994). Due to the significant roles of incumbents and successors in the family firm, their identity work not only affects individual-level consequences, but also results in corporate outcomes, as individual satisfaction and firm performance can be improved when they accept each other's identity in succession (Sharma et al., 2001; Huang et al., 2020). From this point of view, the concept of identity provides an explanatory mechanism for understanding family business succession, that is, when faced with different tasks at different phases of succession, the incumbents and successors behave according to corresponding foci of identity perception. The switching and redefinition of identity will facilitate the sense of belonging and succession behaviors; otherwise, intergenerational conflict emerges and succession suffers. In recent years, studies have introduced the concept of “identity” at various levels to explain the phenomenon within the family firm (Bettinelli et al., 2022), as well as individual identity problems during succession (Le Breton-Miller and Miller, 2014). As the stage where identity issues are more acute, the period of succession is more likely to bring identity challenges. To provide a comprehensive overview of the challenges of identity perception and identity work during succession, this article reviewed 99 SSCI-indexed papers, and raises the following three research questions to analyze the application of identity concept in succession:

**Q1:** How does the concept of identity explain succession behaviors at different succession stages?
**Q2:** What are the antecedents and consequences of identity perception? Which variables can be moderators?
**Q3:** What other important issues at the individual level have been overlooked in family business succession research and motivate future research?

To answer Q1 and Q2, this article takes identity perception and identity work of the sen-gen and the jun-gen as an entry point, and draws on the identity-based perspective, including social identity theory (SIT) and role identity theory (RIT) (Mead, 1934; Tajfel, 1981) to code and analyze all 99 articles, and focuses on the evolution of identity perception and succession behaviors. Specifically, this article refers to Le Breton-Miller et al. (2004) classification of succession phases and tasks, groups 99 articles into nine categories based on the content and subject of each article, and finally proposes a knowledge framework to summarize a general identity mechanism in succession. To answer Q3, this article compares the research gaps and future directions proposed in the sample article, refers to key concepts in identity theory and CEO succession literature, and proposes future research directions around the research topic, perspective, and method based on the framework mentioned in Q1.

The theoretical and practical implications of this review are as follows. First, by introducing the concept of identity, this review comprehensively explains identity perception and identity work mechanism at different phases of succession, and at the same time, the proposed knowledge framework provides future directions on family business succession research. Second, the integrated model can help family business members or advisors to formulate succession planning in advance and recognize identity problems in succession practice. Third, this article emphasizes that succession is not a linear process, but rather a cyclical process of intersubjective continuous feedback and negotiation, and explains the interactive mechanisms. Finally, the identity perspective proposed in this article also helps to explore the psychological mechanisms of CEO succession in non-family, and leadership succession can be better understood from the dynamic and interactive nature of identity construction.

## 2. Identity theories

Identity studies have described key processes of identity formation, activation, and behavior. The process through which individuals take themselves as objects and categorize themselves in line with specific social foci constitutes self-identity or identification (Stets and Burke, 2000). Once individuals acquire specific identities, they will intentionally act in ways consistent with the external norms and institutions of the groups to which they believe they belong (Ethier and Deaux, 1994). Identities are created in social contexts, and through the process of identity work, individuals are allowed to manipulate their identities intentionally and, consequently, modify their behaviors (Alvesson et al., 2008). Identity has become a commonly studied topic in management research, but there is considerable variability in the conceptual meaning and theoretical root of identity concepts (Stryker and Burke, 2000). Among them, two different but interrelated strands are social identity theory (SIT), rooted in psychology tradition, and role identity theory (RIT), rooted in sociology tradition (Table 1 summarizes these two theories). Correspondingly, the incumbents' and the successors' behaviors are combined results of the two identity perceptions. In particular, SIT argues that individuals' categorization process focuses on group membership, so that self-concept derives from in-group similarities, such as belonging perception to the corporate group, and responsibility and obligation to the family group (Ashforth and Humphrey, 1993). In contrast, RIT believes that individuals' self-concept derives from the roles they play in a structured society and the behaviors they adopt to fulfill that role expectation in an interaction relationship (Katz and Kahn, 1978), such as teacher–student roles and leader-subordinate roles.

**Table 1:** Comparison between SIT and RIT.

	**SIT**	**RIT**
Similarity	Identity is an individual's understanding of self-concept, and identification with a particular group leads to behavior.	
Focus of identification	Membership in a group.	Position in hierarchical structures and social relation.
Source of identification	Group value.	Role expectation with the counter role.
Formation process	Individuals categorize themselves according to the group value, and then confirm in-group identification.	Individuals acquire self-concept in interaction, and then confirm role identification.
Explanation of succession	Succession intention originates from an individual's identification with the family and the firm group.	Succession behaviors and role conflicts originate from an individual's parent-child role and leader-future role.

On the one hand, SIT believes that an individual's self-concept derives from membership in a social group, and gains similar values and emotions (Tajfel, 1981). In the SIT theoretical context, the focus of cognitive classification is on group value, and through group and intergroup categorization, actors regard themselves as group members and perceive, think, and behave in similar ways (Ashforth and Humphrey, 1993). SIT, therefore, emphasizes in-group coherence, that is, shared group interests and concerns (Stryker and Burke, 2000), which further results in beneficial decisions and behaviors (Monroe, 2001). In management studies, SIT-motivated studies consider members' turnover decisions, extra-role, and sacrificial behavior as the consequence of commitment and group identification. Correspondingly, in family business succession, the identification with the family and the firm group of the two generations is particularly critical, and the psychological factor of group affiliation is often introduced to explain their succession intentions, such as intra-family successor choice, and career choice in the family firm. It is worth noting that some of the sample articles adopted the term “organizational commitment” or “affective commitment” to explain succession intention, but the argumentation remains the concept of self-identity and identification. As the aim of this article is on the succession process, we did not differentiate between “organizational commitment” and “organizational identification,” for a detailed discussion of the two concepts, see Ashforth et al. (2008, p. 332).

On the other hand, RIT derives from symbolic interactionism (Mead, 1934), and pays more attention to the inter-role interactive norms attached to one's role identity, believing that individuals' self-concept derives from the roles they play in a structured social group (Mead, 1934; Stets and Burke, 2000). In the RIT theoretical context, the focus of cognitive classification is on the position in hierarchical structures and social relations, emphasizing relations with the counter role, and maintaining social structure through interrelated meanings, expectations, and resources (Stets and Burke, 2000). RIT is therefore concerned with relativity and uniqueness, whereby actors regard themselves as unique entities, distinct from other members of the group, and act in accordance with role expectations. Role expectation refers to the idealized standard of a specific role should behave, including personality traits, beliefs, and behavior style (Katz and Kahn, 1978). Once the role identity is activated, individuals will improve their self-efficacy to maintain this role (Stets and Burke, 2000). Role identity regulates one's behaviors, such as leader identity. Whether individuals are designated leaders depends not only on whether they can incorporate the leader identity into their self-concept but also on whether their characteristics and behaviors fulfill the followers' expectations (DeRue and Ashford, 2010). Nevertheless, leadership, as an identity, is ambiguous and susceptible to social construction, as there is no clear or acknowledged standard (Bass and Bass, 2008), and self-perception of the leader identity can vary in strength and is often confronted with legitimacy and authority issues (Miscenko et al., 2017). The extant literature has found that the progress of leadership succession heavily depends on the construction of the leader identity of the candidate (Miscenko et al., 2017). Correspondingly, in family business succession, the identification with the leader role of the two generations is particularly critical, so studies introduce leader identity to explore succession behaviors, such as handover and foster behaviors, as well as takeover and learning behaviors.

**Figure 1:**
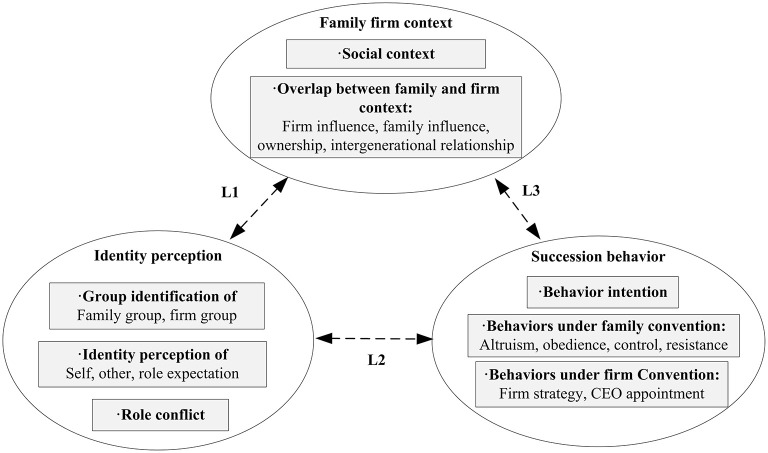
“Family firm context—identity perception—succession behavior” analytical framework.

On the whole, identity theories explain how incumbent and successor produce succession intention and behavior: they categorize themselves in line with specific social foci and then generate identity perception and motivation, which further form succession behaviors to change the context. This mechanism provides new insight into the succession process. As shown in Figure 1, the sen-gen and the jun-gen develop identity perception according to their understanding of the family group, firm group, and the leader role [as shown in Line 1 (L1)], that is, whether they belong to that group or are able to meet leader role expectations. This identity perception further motivates succession behaviors that benefit the family and firm group, or confirm leader role expectation (L2), which in turn, influence the family or the firm context (L3). As succession behaviors process, the incumbent's and the successor's identity perception changes (L2), not only in the evaluation of each other and of the self-concept, but also in the change of identity foci. In this way, the two generations drive succession from the initial phase to the end. In line with this identity concept, this article aims to review family business succession literature at different phases to clarify the identity mechanism of succession task and topic, and summarize a knowledge framework to incorporate antecedents and outcomes, thus providing a sophisticated picture to aid in understanding identity concepts in succession.

## 3. Methods

### 3.1. Article selection and coding process

**Figure 2:**
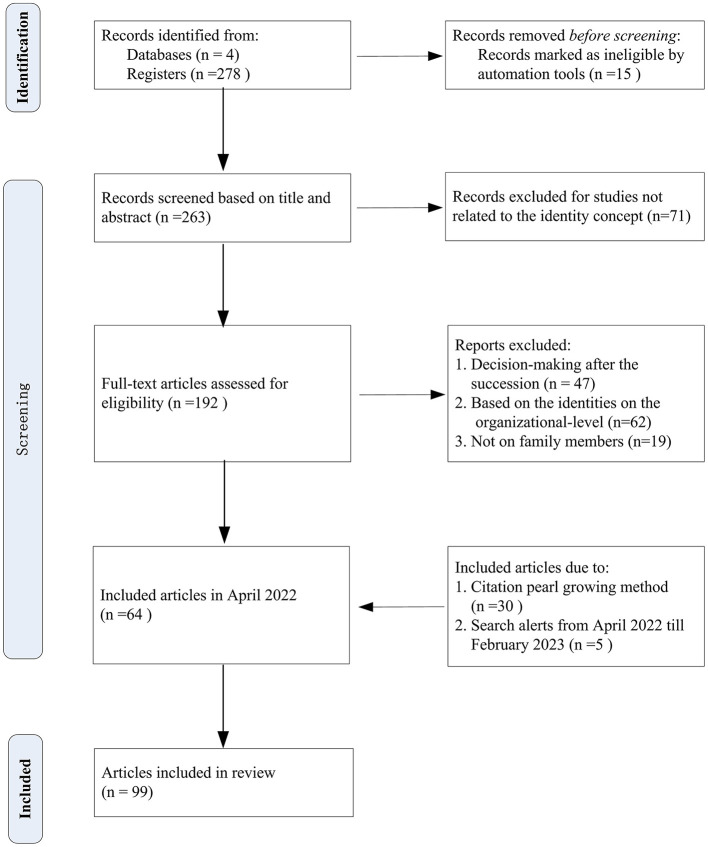
Summary of article collection and screening, adapted from PRISMA.

To comprehensively review the individual-level of identity concepts in family business succession, and discover the relationship between identity perception and succession behavior, this study followed Reporting Items for Systematic Reviews (PRISMA) in article collection, screening, eligibility, and final selection. Figure 2 presents the PRISMA checklist (Page et al., 2021) in each step.

#### 3.1.1. Identify the research objective and identity boundaries

This article aims to review the incumbents' and the successors' identity perceptions, as well as the subsequent succession behaviors. That is, this article incorporates the identity concept at the individual level, rather than identities on the firm level, so that articles on corporate identity are excluded, such as studies on familiness (e.g., Botero et al., 2013) and branding (e.g., Casprini et al., 2020) of the family firm. Second, this article mainly focuses on the key participants, such as the incumbent, the successor, and family members in succession, omitting non-family members, such as non-family managers or advisors (e.g., Härtel et al., 2010).

#### 3.1.2. Article collection

Two authors set the topic words and conducted the first round of searches in April 2022. To ensure comprehensiveness, the Web of Science, EBSCOhost database, Scopus, and Google Scholar were included. The topic words used to search for family business studies include *family business* and *family firm*; the keywords used to search for the succession literature include *succession, successor, next-generation*, and *incumbent*; and the keywords used to search for identity concepts include *identity, identification, identify, role*, and *self/selves*. The search scope includes all available years (including online first) before April 2022. The type of literature was set to “articles.” Two hundred and seventy-eight articles were collected, among which 15 articles were excluded as they were not studies on family business, so the initial sample of 263 articles was obtained.

#### 3.1.3. Article screening

After obtaining the initial samples, the three authors held a meeting to discuss the differences in article content, further clarified the connotation of the concept of identity, and developed exclusion criteria (as shown in Step 1). Then, the authors screened the articles separately and discussed whether to exclude when met disagreement. Specifically, due to the ambiguity of the word “role” in the search term (including the “role identity” meaning and “effect” meaning), the authors removed 71 articles irrelevant to the concept of identity. Further, to concentrate on the identity of the incumbent and the successor, 47 on corporate decision-making after succession, 62 on familiness and branding, and 19 on non-family members were removed, and 64 papers were retained. Next, to prevent omissions, we adopted the citation pearl growing method, namely, by browsing the reference and citing articles of the above 64 articles, we identified another 30 articles which did not include the identity concept in the title or abstract but adopted the identity perspective in the main body of the article (e.g., Frederik and Riar, 2022). Last, the authors re-conducted the second round of search in February 2023, adding 5 articles, and finally, 99 articles were selected as sample articles.

#### 3.1.4. Article assessment and coding

In the assessment, the review considered the quality of journals and the methodology of each article. Most of the sample articles were published in Q1 and Q2 journals, and a few published in Q3 and Q4 are more targeted, such as agribusinesses or region-specific studies, which can broaden the application of identity theories, and therefore are included in the sample. On the other hand, the sample articles in the early years were not rigorous in method or argumentation, but with diachronic comparative significance. In the coding process, the basic information about each article, including the author, publication year, journal, method, and sample source is recorded. Second, we extracted the research topic and identity-related concepts, such as participants, the strategy of succession, theoretical basis, identity perception, and identity work, the antecedents and consequences of identity perception, and the future directions proposed in each article (see Supplementary material for details of the coding results).

### 3.2. Description of reviewed articles

**Figure 3:**
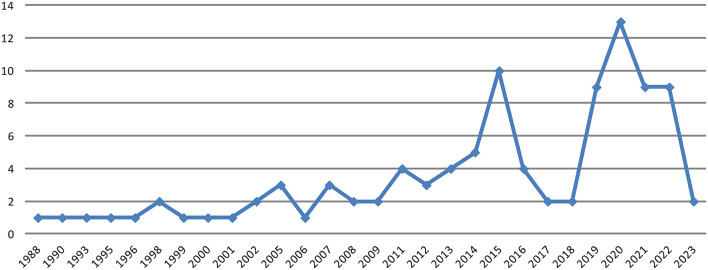
Number of articles by year.

As shown in Figure 3, the publication year of the articles suggests that the identity perspective displays a marked increase in recent years, and for the articles that have not been published, we counted by online publication year. The research can be traced back to Barnes (1988), who first notices hierarchical incongruence between the family and the firm roles of the successor during succession, especially for daughters and younger sons. To construct a leader identity, the successor has to change the incumbent's expectations and perceptions of the child. Two years later, Handler (1990) proposed the well-known mutual role adjustment model between the incumbent and successor, emphasizing their career position mutual adjustment during the succession process, which is highly cited in family business succession study. These two studies provide an early exploration of the identity perspective and lay the foundation for identity's psychological and interactive nature in succession studies. The number of articles remained stable before 2015, and in 2019, the number sharply increased. This may be because the family business research began to explore succession issues from the micro-foundation (De Massis and Foss, 2018), which has become a research trend in recent years.

Regarding the published journals, more than half of the articles are published in journals on family business, small business, or entrepreneurship (as shown in Table 2), while other articles are published in the field of management and economics. And most journals are Q1 and Q2 SSCI ranked, suggesting that although the identity perspective is not superior in quantity, it can reveal the internal psychological mechanism of the participants, so the articles are usually of high quality and receive attention in family business research. At the same time, the rest of the literature is scattered, including journals in economics, psychology, education, industrial psychology, etc. This is because the study of family firms from an identity perspective exhibits cross-disciplinary features, attracting interest from many research areas with the potential to make an interdisciplinary contribution.

**Table 2:** Number of articles on the identity perspective per journal (>1 article).

**Journal**	**Number of articles**	**Cumulative frequency (%)**	**Rank in SSCI**
Family Business Review	23	23.2	Q2
Entrepreneurship Theory and Practice	17	40.4	Q1
Journal of Family Business Strategy	11	51.5	Q2
International Small Business Journal	5	56.6	Q2
Journal of Small Business Management	4	60.6	Q2
Sustainability	4	64.6	Q2
Administrative Science Quarterly	2	66.7	Q1
Frontiers in Psychology	2	68.7	Q1
International Journal of Entrepreneurial Behavior and Research	2	70.7	Q2
Journal of Business Research	2	72.7	Q1
Small Business Economics	2	74.7	Q1
Total	74	74.7	**–**

Among all 99 reviewed articles, 45 articles adopted inductive logic to formulate propositions or conclusions through case studies or empirical observations; 6 adopted a mixed method, where hypotheses were first formulated through a case study and then tested by data. The other studies used inductive logic: 29 articles tested the hypothesis through questionnaires, 11 were conceptual articles, and 8 tested the hypothesis through panel data. This indicates that the identity-based perspective mainly explored “why” and “how” questions in succession with case studies, regarding identity as a process, providing insights into the incumbent's and the successor's psychological antecedents and the dynamic interaction in identity work. On the other hand, deductive studies regarded identity as invariable property, testing the relationship between identity perception and behavior tendency with a questionnaire or panel data. It is worth noting that as a questionnaire can measure participants' psychological and cognitive characteristics, it becomes the second most commonly used method in identity studies. In contrast, panel data are seldom used, as it is difficult to seek an appropriate variable to represent the identity concept developed by the author.

In terms of research context, most studies chose family businesses in Europe (45 in all, including 10 in Germany, 7 in Italy, 6 in Spain, and 5 in France, etc.), North America (24 in all, including 19 in the USA and 5 in Canada), and Asia (14 in all, including 9 in China, etc.), while other 4 articles collected questionnaires from Africa, South America, and global research. Family businesses in Europe and the US have a large research sample for the case and questionnaire research as they have experienced multiple generations, which also provides opportunities for retrospective, diachronic research. And as family firms in Asia enter the peak of their first succession, a new research trend is beginning to grow within China as the study context. And as mentioned in the discussion section, future research is supposed to analyze identity norms and behavioral standards in different cultural contexts through cross-cultural comparative studies.

## 4. Results

### 4.1. Research topic and categorization of the sample article

After reviewing the research topics of all 99 articles, we find that the three-phase model proposed by Le Breton-Miller et al. (2004) can better explain succession phases and the correspondent succession tasks, as well as related identity concepts. Hence, we categorize sample articles according to the succession phases and research object. Specifically, 99 sample articles were divided into nine categories based on three succession tasks (as shown in Table 3), namely, “succession intention,” “successor nurture,” and “power transfer”; and three types of research perspectives, namely, “incumbent perspective,” “successor perspective,” and “interactive perspective of the two generations.” The articles without a clear statement of the phase were categorized by browsing the background of the study and the succession tasks included in the study. However, as articles may discuss succession from both incumbent and successor perspectives and case studies usually cover several phases (e.g., Handler, 1990), some articles may be classified into two categories.

**Table 3:** Succession topic, identity concept, and categorization of sample article in three phases.

	**Phase 1: Intra-family succession intention**		**Phase 2: Successor nurture**		**Phase 3: Power transfer**	
Succession topic/Frequency	Takeover intention (24), successor choice (11), career (9), gender (8), commitment (7), transgenerational intention (6), turnover intention (1)		Foster and development (18), recognition (7), legitimacy tactics (5), successors' strategic decision (6), gender (5), entrepreneurship (4), birth order (3), control (2), autonomy (1), opportunity perception (1), leadership skill (1)		Mutual acceptance (6), contradictory emotion and behavior (5), retention or retirement (3), discretion (2), clear boundary (1)	
Identity concept	Identification with the family group or firm group, leader role as motivation, expectation of the leader role		Leader identity work, self-perception of leader identity, leader type, expectation of the leader role		Role conflict, identity confirmation, relinquish leader role, leader role as motivation	
Articles from incumbent perspective	Ahrens et al., 2015 Alterman et al., 2020 Aygoren and Nordqvist, 2015 Calabrò et al., 2018 Chen et al., 2021 Chrisman et al., 1998 DeNoble et al., 2007 Fang et al., 2023	Jaskiewicz et al., 2016 Lu et al., 2021 Mahto et al., 2019 Mahto et al., 2021 Meroño-Cerdán, 2022 Sharma and Srinivas Rao, 2000	Barbera et al., 2015 Cabrera-Suárez, 2005 Cadieux, 2007 Canovi et al., 2022 Foster, 1995 García-Álvarez et al., 2002	Hauck and Prügl, 2015 Jaskiewicz et al., 2015 Martínez-Sanchis et al., 2020 Salvato and Corbetta, 2013 Samei and Feyzbakhsh, 2015 Shanine et al., 2022	Cadieux, 2007 Frederik and Riar, 2022 Gagné et al., 2011	Huang et al., 2020 Lam, 2011 Sharma et al., 2001
Articles from successor perspective	Björnberg and Nicholson, 2012 Bloemen-Bekx et al., 2021 Cabrera-Suárez and Martín-Santana, 2012 Cabrera-Suárez, 2005 Chalus-Sauvannet et al., 2016 Chlosta et al., 2012 Curimbaba, 2002 Dawson et al., 2015 Eckrich and Loughead, 1996 Feldmann et al., 2022 Gimenez-Jimenez et al., 2021 Radu-Lefebvre and Lefebvre, 2016 Mahto et al., 2020 Martin-Cruz et al., 2020	McMullen and Warnick, 2015 Murphy and Lambrechts, 2015 Murphy et al., 2019 Overbeke et al., 2013 Porfírio et al., 2019, 2020 Romaní et al., 2022 Schell et al., 2020 Schröeder et al., 2011 Schröder and Schmitt-Rodermund, 2013 Sharma and Irving, 2005 Sharma et al., 2001 Stavrou and Swiercz, 1998 Wielsma and Brunninge, 2019 Xian et al., 2021 Zhu and Zhou, 2022	Ahrens et al., 2019 Bika et al., 2019 Byrne et al., 2021 Cabrera-Suárez, 2005 Calabrò et al., 2018 Cater and Justis, 2009 Chen et al., 2021 Dalpiaz et al., 2014 Frederik and Riar, 2022 Garcia et al., 2019 Goldberg and Wooldridge, 1993 Haberman and Danes, 2007 Hytti et al., 2017 Kandade et al., 2021	Lauto et al., 2020 Le Breton-Miller and Miller, 2014 Leotta et al., 2017 Mair and Rombach, 2020 Miller, 2014 Mitchell et al., 2009 Mussolino and Calabrò, 2014 Mussolino et al., 2019 Pruthi and Tasavori, 2022 Sardeshmukh and Corbett, 2011 Schenkel et al., 2016 Torres et al., 2023 Vera and Dean, 2005 Wang and Zhang, 2022 Yoo et al., 2014	Barnes, 1988 Lauto et al., 2020 Querbach et al., 2020	Radu-Lefebvre and Randerson, 2020
Articles from interactive perspective	Byrne et al., 2019 Overbeke et al., 2015		Handler, 1990 Matthews et al., 1999 McAdam et al., 2021		Ahrens et al., 2018 Bertschi-Michel et al., 2020 Cooper et al., 2013 Klein, 2008	Li and Piezunka, 2020 Milton, 2008 Venter and Boshoff, 2006

As shown in Table 3, the initial task in phase 1 is to address succession planning, so that the corresponding research topics focus on intra-family succession intentions, including the incumbent's intention to keep the firm in the family and preference for successor choice, as well as successor's takeover intention. Drawing on SIT, these two types of intentions can be traced back to the sense of obligation for family continuity triggered by the identification with the family group, and jun-gen's identification with the firm group. Meanwhile, the expectation of the leader role prompts the incumbent to choose the offspring with certain characteristics as successor, and the jun-gen to join the family firm as a future career choice. Phase 1 ends up with the successor entering the family firm and the succession process begins.

Phase 2 addresses the successor training, so that the corresponding research topics focus on leadership development, incumbent acknowledgment, entrepreneurship and opportunity perception, and decision-making. From the RIT perspective, the above-mentioned nurture behaviors stem from the incumbent's role expectation of the leader and following formal or informal leader construction behaviors. And the decision-making stems from the successor's motivation to engage in identity construction in order to meet self-expectations, incumbent expectations, and stakeholder expectations.

Phase 3 addresses the power transfer issue when the family firm is faced with a situation where two leaders share governance. So that the corresponding research topics focus on incumbent retirement, acknowledgment, role conflict, and related contradictory emotions. From the RIT perspective, the perceived role conflict triggered by the overlap between family and firm context often leads to ambivalence and difficulties in succession, as both sen-gen and jun-gen are struggling between family authority convention and firm autonomy convention. Succession can only run smoothly when the incumbent and the successor are able to manage role conflicts.

### 4.2. Reinterpretation of succession from the identity perspective

To elaborate on the reinterpretation of succession topics with identity concepts, and explain the mechanisms by which identity perception generation succession behavior, the following section will adopt the analytical framework of “family firm context—identity perception—succession behavior” proposed in Figure 1, clarify each of nine categories delineated in Table 3.

#### 4.2.1. Phase 1: Intra-family succession intention

The main task in phase 1 is to develop a plan, i.e., the initial determination of intra-family succession intention (Le Breton-Miller et al., 2004). Sample articles address the sen-gen and the jun-gen as cognitive subjects individually. On the one hand, for the study of the sen-gen, SIT argues that the incumbent's sense of responsibility toward the family group exposes pressure for family continuity, that is, when the incumbent ties self-concept closely to the family group, a strong sense of closeness, reciprocity, and commitment to the family develops. And to maintain family prosperity, s/he chooses the family member as the successor to continue family control in the business (Jaskiewicz et al., 2016; Mahto et al., 2019). This situation is evident in more traditional (Lu et al., 2021) or deeply influenced by Confucianism incumbents (Chen et al., 2021), who prefer to maintain a respected position in the family (Alterman et al., 2020). In contrast, incumbents with lower family continuity pressure may prioritize business interests and prefer a professional manager rather than a family successor (Mahto et al., 2019), also, they are easy to be persuaded by advisors to choose a non-family successor (Mahto et al., 2021). For example, female leaders (Aygoren and Nordqvist, 2015) and incumbents who have experienced multiple generations of succession (Calabrò et al., 2018) usually have a lower sense of identification with the family group and a lower intra-family succession intention.

Further, according to RIT, incumbents have specific role expectations of the future firm leader, which result in preference in the candidate pool (Fang et al., 2023). Although early research suggested that in successor selection, incumbents usually emphasized ethical criteria, such as integrity and commitment to the business (Chrisman et al., 1998), or interpersonal capability and experience (Sharma and Srinivas Rao, 2000), recent research has found that incumbents' leadership criteria exhibit masculine traits, such as risk-taking spirit (Byrne et al., 2019), primogeniture, or male succession (Ahrens et al., 2015; Calabrò et al., 2018). However, this criterion of pan-masculine traits breaks down when faced with performance below expectation (Calabrò et al., 2018; Meroño-Cerdán, 2022) or when daughters have higher human capital (Ahrens et al., 2015).

On the other hand, for the study of the jun-gens, their identification with and commitment to the family business are important predictors of the decision to pursue a career in the family business and construct a leadership identity (Sharma and Irving, 2005; McMullen and Warnick, 2015). Ashforth et al. (2008) distinguished between the concepts of organization identification and organization commitment, emphasizing that the former results in the attachment of organization membership with self-concept, while the latter does not modify self-concept, but is concerned with satisfaction with the organization. However, as family business studies mainly focus on affective commitment, they have not clearly distinguished between these two concepts, and articulate the logic of the successors' self-identity with the term “commitment” when analyzing succession intention. In this case, the antecedents of successor commitment to the family business can be traced back to the perception of self-concept with the family group, the business group, and the leader identity (Björnberg and Nicholson, 2012), forming a research path of “identity perception—organization commitment—succeed intention” (Dawson et al., 2015; Gimenez-Jimenez et al., 2021). Specifically, jun-gens' perception of the surrounding group motivates them to make choices that are beneficial to the group, so that when the jun-gen connects the self-concept with the firm group, the intergroup consistency strengthens his/her desire to join and contribute to the family firm (Schröeder et al., 2011; Schröder and Schmitt-Rodermund, 2013; Bloemen-Bekx et al., 2021; Lu et al., 2021; Romaní et al., 2022). Herein, jun-gens' affective commitment makes the best contribution to successful succession and efficient management of the family business (Sharma and Irving, 2005; Cabrera-Suárez and Martín-Santana, 2012). To this end, research has suggested that identification with the family firm can be improved through deliberate tactics, such as early involvement and internship (Murphy et al., 2019; Gimenez-Jimenez et al., 2021), family story-telling (Bloemen-Bekx et al., 2021), and reinterpretation of enterprise mission to reconstruct the company image (Wielsma and Brunninge, 2019; Sasaki et al., 2020). However, what calls for attention is that jun-gens' early participation can lead to stereotype and path dependence, resulting in poor business performance after succession (Ahrens et al., 2019).

Further, according to identity theory, different dimensions of identity can be simultaneously salient when motivating a behavior (He and Brown, 2013). Motivated by RIT, successors' expectations and planning of the leader identity constitute the motivation to join the family firm (Schröder and Schmitt-Rodermund, 2013; Radu-Lefebvre and Lefebvre, 2016; Porfírio et al., 2019, 2020). Taking over the business means choosing the firm leader role as the future career for the successor, which requires making decisions in a complex environment and brings challenges. Social constructivists regard the successor's sensemaking during succession as an intersubjective phenomenon (Fuller and Loogma, 2009), that is, his/her perception of becoming a firm leader comes from the interaction before the succession process, especially from perceived familial and social expectations (Chlosta et al., 2012). For example, the stereotypes based on gender, age, and education in the hierarchy and social norms lead daughters and younger sons to often ignore the possibility of their future succession (Curimbaba, 2002; Overbeke et al., 2013; Byrne et al., 2019; Xian et al., 2021). Of course, such identity perception is not rigid, but exhibit dynamic feature in intergenerational interaction. For example, a clear succession plan (Porfírio et al., 2019, 2020), the incumbent's training behavior, the role model (Chlosta et al., 2012; Feldmann et al., 2022), support (Zhu and Zhou, 2022), control (Eckrich and Loughead, 1996; Schröder and Schmitt-Rodermund, 2013), and successor's learning behavior, such as educational experience and internship (Chalus-Sauvannet et al., 2016), and imprinting from childhood life (Murphy and Lambrechts, 2015) can improve the offspring's self-efficacy to assume the leader role in the future and thus generate takeover intention.

In conclusion, role intentions and identity perceptions determine the way in which the incumbents and the successors behave. Forming a specific identity perception becomes significant in phase 1. On the one hand, the self-concept of the incumbents, that is, the family responsibility motivates them to prepare the family member as a successor. On the other hand, identity perception within the family group as well as leader role intention is the prerequisite for succession. In this way, the subjective perception of both the incumbents and the successors stimulates their succession intention and motivation, which constitute the premise of family business succession.

#### 4.2.2. Phase 2: Successor nurture

For the candidate who has acquired a family firm identification, a successful succession depends on the successor's self-construction of leader identity with the assistance of the incumbent, as well as further recognition from the stakeholders, which constitutes two dimensions of the identity work in phase 2. The successor-centric studies focus on the candidate's psychological and cognitive construction of the self-expected leader identity and further, how s/he can be acknowledged by stakeholders. First, RIT believes successors themselves hold a set of role expectations of the leader, such as organizational skills, personality traits, and interpersonal skills (Mair and Rombach, 2020). Influenced by the leader role expectation, they develop a sense of self-efficacy toward the leader role, which becomes the internal motivation to generate leader identity work, as only when successors believe they can assume the leader identity will they display leader/entrepreneur behavior and deepen involvement in the family firm (Garcia et al., 2019), which in turn increases intergenerational satisfaction with the succession (Cabrera-Suárez, 2005). Motivated by this cognition-behavior mechanism, the successor's growing experience, and the incumbent's nurturing behavior constitute the key context for the successor's self-concept: successors' life experience, such as internship experience (Murphy and Lambrechts, 2015), exposure to family business-specific knowledge, tacit knowledge, observation, and imitation (Sardeshmukh and Corbett, 2011; Chen et al., 2021) help them to improve self-efficacy specific to the family business.

Second, incumbents' nurture, such as resource support (Garcia et al., 2019; Torres et al., 2023), learning by trial and mistake (Cabrera-Suárez, 2005; Haberman and Danes, 2007; Canovi et al., 2022), entrepreneurship development (García-Álvarez et al., 2002; Jaskiewicz et al., 2015), whole-person development (Barbera et al., 2015), expectation communication (Martínez-Sanchis et al., 2020), and authorization (Dawson et al., 2015; McAdam et al., 2021) will improve successor's self-concept (Garcia et al., 2019) and psychological function (Shanine et al., 2022) as leader identity. On the contrary, the incumbent's high authority (Hauck and Prügl, 2015) or excessive control (Garcia et al., 2019), and dysfunctional intergenerational relationships (Miller, 2014; Wang and Zhang, 2022) are likely to result in successors' lower self-evaluation in the firm. Therefore, the incumbent needs to demonstrate openness, trust, and patience in the construction of the successor's leader identity (Samei and Feyzbakhsh, 2015). However, research on successor socialization has reminded that jun-gen nurture should not be limited to intergenerational interaction, but include teams, advisors, non-family managers, and external stakeholders. And under the rapidly changing external environment, incumbents are equally faced with the demands of learning and will benefit from re-socialization.

Third, from the dimension of stakeholder recognition, the successor needs to exhibit specific leadership behaviors to fulfill the external expectation. For example, successors often claim leader identity and internalize their management vision through strategic actions, such as innovation or entrepreneurial strategies (Hauck and Prügl, 2015; Frederik and Riar, 2022), and internal management practices, such as management accounting and employee incentive programs (Leotta et al., 2017; Shanine et al., 2022). Further, the successors interact with employees and external stakeholders through narrative strategies to highlight the family, including constructing a sense of family, celebrating family achievements, and emphasizing non-family member endorsement (Dalpiaz et al., 2014). Apart from symbolic strategies, successors adopt interactive strategies to develop a relationship with non-family members and legitimacy as the future leader in the family business, such as mutual respect, trust, obligation, early involvement, and mentoring (Kandade et al., 2021).

It is noticeable that studies often consider characteristics such as gender, age, and personality as the proxy for role expectations. For example, incumbents tend to identify and define daughters' firm roles based on family roles (Vera and Dean, 2005), thus often ignoring their possibility to lead the business (Hytti et al., 2017), or granting assistant positions (Xian et al., 2021). In this scenario, the daughters have to construct a recognized leader identity through intergenerational interactions and, to a greater extent, interactions with employees to negotiate their original expectations of the leader role (Mussolino et al., 2019; McAdam et al., 2021). Similarly, birth order has also been considered a determinant of leader identity construction (Bradley, 1982), with second sons less likely to be leaders of the family firm than the eldest (Yoo et al., 2014; Schenkel et al., 2016), making it more difficult to construct firm roles. However, the family characteristics of successors imply that they have different upbringing and development experiences, which lead to a difference in leader identity internalization and decision-making. For example, once non-first-born son succeeds, as he has greater psychological distance and prefers uniqueness, their decisions usually focus on corporate interests rather than family interests (Calabrò et al., 2018), resulting in the appointment of non-family members as executives and better corporate performance (Schenkel et al., 2016). Moreover, Pruthi and Tasavori (2022) further analyzed how strategy differentiates immigrant successors, namely, how firm growth strategy is influenced by successors' ethnic ties and family ties.

In all, phase 2 concerns internalizing the leader role, and although the successor may already be in the leader position, it is necessary to develop the self-efficacy of the leader and exhibit leader behaviors. Specifically, in phase 1, the successors have initially formed leader role intention and guide selves in succession tasks, however, it is clear that role intention cannot prepare them as firm leaders. The incumbents and the successors rely on a series of nurturing and learning behaviors, and through these shared experiences they develop a leader identity perception of the successor, which becomes the core of phase 2.

#### 4.2.3. Phase 3: Power transfer

As the successor constructs the leader identity, the existence of two leaders in the family business leads to the incumbent's contradictory behaviors between empowerment and domination, and the successor's ambivalent emotions of autonomy and compliance, which can be traced back to role conflict in the family business. That is, the overlap between the family and the business groups suggests that the sen-gens and the jun-gens need to follow two role norms during the succession (Cooper et al., 2013; Li and Piezunka, 2020). This contradictory role perception intensifies in the later stages of the succession (Bertschi-Michel et al., 2020), which would further lead to intergenerational conflict, poor succession, conservative decision-making, and even threaten business performance (Sharma et al., 2001; Klein, 2008; Milton, 2008; Ahrens et al., 2018; Querbach et al., 2020). As a result, family businesses often consult non-family managers or advisors (Handler, 1990; Bertschi-Michel et al., 2020), and spouses of the incumbents (Li and Piezunka, 2020) to set the boundary between the family and the firm and clarify behavior norms in succession (Bertschi-Michel et al., 2020; Li and Piezunka, 2020).

Studies analyzed role conflict in phase 3 individually from the sen-gen and the jun-gen. On the one hand, the incumbent's contradictory behaviors originate from the structural antecedent of multi-roles (Lam, 2011; Li and Piezunka, 2020). In phases 1 and 2, the sen-gen, as the parent in the family, is proud of the children's entry into the family firm and their outstanding achievements, and is willing to “let go” (Lam, 2011). However, in phase 3, when concerning actual handover decisions, the incumbent is unwilling to give up the leader identity, though still holding intra-family succession intention. This is because, besides the parent in the family, the sen-gen is also the leader of the firm and takes responsibility for the firm performance; thus, s/he becomes critical of the successors' competency and considers them “too young” to take over the business. Moreover, in pursuit of self-concept congruence (Frederik and Riar, 2022), the incumbent is unwilling to relinquish the leader identity, as s/he tightly attaches his/her self-concept to hero roles and missions (Sonnenfeld, 1988). That is, the more significant the incumbent perceives leader identity to self-concept, the more negative s/he will be when retiring from the business (Gee, 1999). And the incumbent's characteristics, such as goal-adjustment ability (Gagné et al., 2011) and narcissistic traits (Huang et al., 2020), determine his/her ability to distinguish between role conflicts and make adjustments to self-concept. Studies also suggested that successor behavior is a key context element for the incumbent's perception of self-concept: when the successor performs autonomous and entrepreneurial, the incumbent perceives a higher level of threat and thus increases control over the successor (Huang et al., 2020).

From the perspective of the successor, when s/he has acquired knowledge and experience of being a firm leader, the incumbent's contradictory behaviors usually lead the successor to ambivalent emotions between compliance and autonomy: when enacting the family role, the jun-gen is obliged to obey the routines that parents have established, especially when the successor is more closely tied to the family and appointed by the incumbent (Querbach et al., 2020). However, when playing the firm role, the successor is supposed to get rid of the incumbent's control and attain autonomy. These two conflicting norms usually cause emotional disorders (including pride, joy, anxiety, and envy), which further aggravate conflicts in succession (Radu-Lefebvre and Randerson, 2020). According to boundary management strategy (Knapp et al., 2013), communication becomes the primary tactic to resolve role conflict (e.g., Eckrich and Loughead, 1996; Knapp et al., 2013). However, the incumbent and the successor alone cannot manage role conflicts, and a third party must be introduced to define the boundary between family and business so that family relationships remain unaffected by business conflicts, such as mothers who do not work in the family business (Li and Piezunka, 2020), trusted advisors (Bertschi-Michel et al., 2020), or friends with high status (Barnes, 1988).

In conclusion, in phase 3, power transfer manifests as a combination of granting and claiming, a process of both successors' leader role perception and incumbents' perception of self-concept. This review emphasizes conflicting identity perceptions in the context of family business succession, that is, the incumbents and the successors face two role norms and identity perceptions due to the overlap between the family and the firm. And this role conflict, which is unique to family firms, will hinder the completion of successors' leader identity.

## 5. Discussion

### 5.1. A knowledge framework

**Figure 4:**
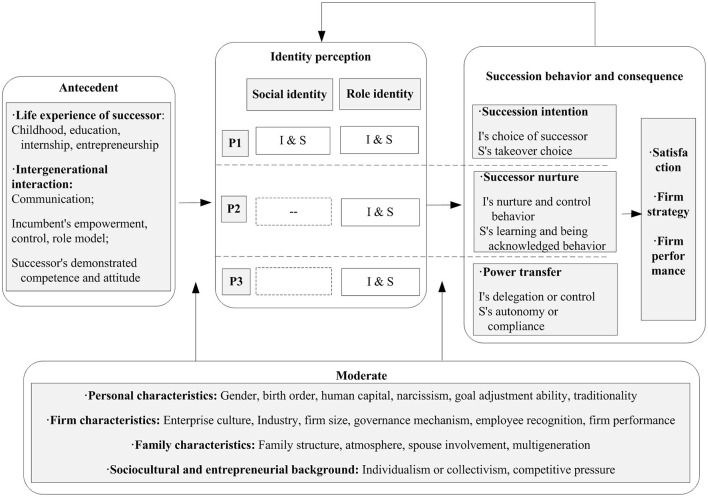
Knowledge framework of succession from the identity perspective.

To answer the first and the second research questions, this study categorizes key variables mentioned in the sample articles, and summarizes a knowledge framework of succession from the identity perspective (as shown in Figure 4), including elements of the antecedent, connotations of identity perception, succession behavior, consequence, and moderate variables. Where “P” refers to succession phases: P1 = Phase 1; P2 = Phase 2; P3 = Phase 3; “I” and “S” refers to the subjects of identity perception: I = the senior generation or the incumbent; S = the junior generation or the successor. Due to the continuity of individual psychology and cognition, the influence of psychological characteristics on succession behavior is intertemporal; that is, the identity perception or behavior in one phase may lead to the succession behavior or firm consequences in the next two phases. As a result, this study integrates all the related variables and possible relationships in three stages proposed by the sample articles into one knowledge framework, which helps us to explore directions for future research.

First, this article summarizes the antecedents that generate identity perception (as shown in the left box and the bottom box in Figure 4). Normally, the immediate context for identity perception stems from upbringing experiences and intergenerational interactions, such as the jun-gen's experience, the sen-gen's behavior, as well as communication and conversation. However, such effects are not uniform or static, in that according to upper echelons theory, the cognitive inclination shaped by personal characteristics, such as gender, human capital, and personality usually leads to different identity perceptions given the same experiences (act as a moderating variable). Further, the context, constituted by the family atmosphere, firm characteristics (represented by firm culture and industry), and social context (represented by culture and business policy) become important external factors that influence identity perception.

Second, this article summarizes the shift of the perception foci, which formulates the identity lens in the succession study (as shown in the middle box and the right box in Figure 4). From the perspective of the sen-gen, his/her foci of identity perception mainly shift from the group role to the leader role and multi-roles. That is, in phase 1, the incumbent's self-concept of the family group and the firm group, as well as the role expectation of the firm leader together, decide the choice of the successor. In phase 2, the incumbent's identity focus turns to the role identity, which motivates their nurture and control behaviors. With the leadership development of the successor, the focus of identity perception turns back to the self-concept of multi-roles, in that the successor's leader identity threats the incumbent's self-concept, which may trigger ambivalence in delegation and control. Correspondingly, from the perspective of the jun-gen, s/he experiences similar identity perceptions. Specifically, in phase 1, the jun-gen's self-concept in the family group and the firm group, as well as the fulfillment of the leader role expectation in the future together decide his/her takeover intention. And this identity motivation extends to phase 2 as a significant origin of leader construction behaviors. In phase 3, the focus of identity perception turns to the multi-roles, leading to the dilemma of autonomy and compliance.

Third, this article emphasizes the behavior consequence and feedback effect of identity perception (as shown in the right box and the middle box in Figure 4). As the succession behaviors of the present phase will not only cause firm consequences, but also trigger emotion and cognition changes in the next phase, forming a continuous feedback loop. Previous studies, like Handler (1990) had suggested the mutual adjustment of roles between the generations. In recent years, this topic has begun to attract attention. For example, in the initial phase, daughters are able to negotiate leader role expectations with the incumbent through identity works and intergenerational interactions (Xian et al., 2021). McAdam et al. (2021) analyzed the dynamics of intergenerational relationships, that is, daughters engage in identity works to gain recognition, while developing independently to construct a leader identity that is recognized by other stakeholders.

### 5.2. Future direction

**Figure 5:**
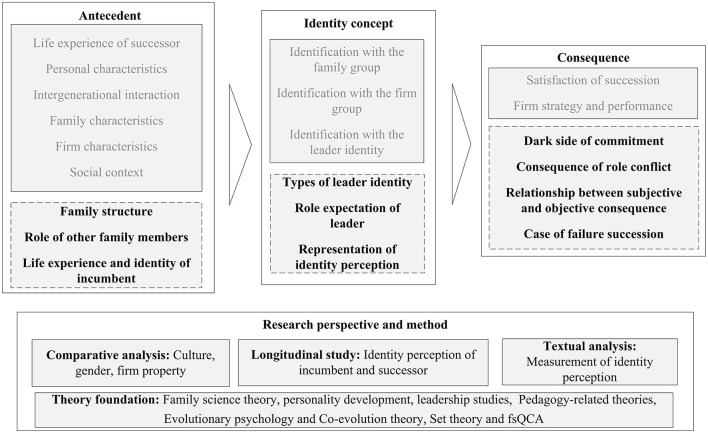
Future directions.

To answer the third research question, this article reviews the research gaps and possible research directions identified in the 99 sample papers, and consults key concepts in identity theory and leadership succession research, and finally proposes future directions based on the existing knowledge framework. As shown in Figure 5, the following will discuss potential research gaps in research topics and research perspectives. The gray font refers to the existing research topic or method, and the bold font refers to the future direction proposed in this study.

#### 5.2.1. Research topics

In terms of the antecedents of identity perceptions, the present studies have explored the proximal context of successor experience, personal and intergenerational elements, as well as the distal context of the family, the firm, and the local characteristics. Future research could further explore the influence of family structure, such as whether children in single-parent families have a stronger identification with the family group and therefore choose to contribute to the family through entrepreneurship. It is also meaningful to analyze the role of other members in the family, such as whether siblings of the successor can alleviate intergenerational conflict, introduce new knowledge, or exacerbate competition awareness in the offspring (Bika et al., 2019). Another interesting question is when considering the incumbent's self-concept, how do the personal experience and cognitive tendency affect identity perception during succession? As the studies have analyzed the impact of the incumbent's gender and personality on identity perception, ignored the effect of life experience. For example, will incumbents accelerate the succession process when they are faced with disease (Alterman et al., 2020)?

On the dimensions and connotations of identity perception and behavior, the existing studies simplify the role adjustment process as “no role—helper—manager—leader” (Handler, 1990), which misses specific role expectations of the leader. For example, drawing on tasks, Mintzberg defines managerial roles as interpersonal roles, information roles, and decision-makers. Similarly, Graf-Vlachy et al. (2020) argue that as tenure advances, the CEO attains role-specific knowledge and a thinking model, suggesting an increase in cognitive complexity. Following the leadership study, this study suggests refining the decision-making behaviors during the whole developmental progress. In addition, future research could explore the habitat of leader identity (Aygoren and Nordqvist, 2015), that is, except for gender, birth order, and education, what criteria are adopted in identity perception? Last, another interesting phenomenon is the symbolic representation of identity perception. According to cognitive linguistics, the choice of appellation reflects the speaker's understanding of the position and power in the relationship (Brown and Gilman, 2012), and the individual's choice of title is influenced by their construal of the circumstance, reflecting their understanding of self and others' identities. Therefore, in the family business, how incumbents and successors address each other reflects their understanding of identity in business. When emphasizing position titles in the firm, they may adapt to business tasks and seldom suffer from role conflict. In contrast, when addressing each other according to a relative appellation system, they usually follow family norms and have trouble constructing incumbents' retirement roles and successors' leader identities.

Concerning the consequence of identity perceptions, studies have concluded that commitment is favorable to succession outcomes, however, Dawson et al. (2015) reminded the dark side of organizational commitment as there may be pitfalls in the successor's commitment. For example, commitment motivated by family interests usually leads to high investment when experiencing losses. Similarly, Murphy and Lambrechts (2015) caution that a successor's attitudes may affect business performance. Second, studies have emphasized multi-roles and succession problems resulting from it. However, apart from ambivalent emotions, researchers have little knowledge of the manifestation of role conflict in succession. This study suggests that future research could explore how role conflict affects power struggles and collusive behavior within the firm. Third, firm succession has to consider the achievement of both corporate and family goals, and when focusing on the subjective and objective consequences of succession, one would wonder about the relationship between them (Sharma et al., 2001). As family members usually make subjective perceptions based on objective performance, in turn, performance is likely to suffer from intergenerational cognitive dissonance, thus creating a mechanism for mutual influence. Last, current research focuses on family firms that have smoothly succeeded, while it would be meaningful to include those failed firms, and consider what factors in the successor may terminate the succession (Ahrens et al., 2015; Radu-Lefebvre and Randerson, 2020).

#### 5.2.2. Research method and perspective

First, the studies under the identity perspective mainly adopted case studies (45), questionnaire research (29), or mixed-method (6), yet almost all of the questionnaire and case studies highlighted self-reporting problems and recall bias, encouraging tracking studies and diachronic analysis to explore changes in intergenerational perceptions and evaluations at different stages. Moreover, there are also epistemological differences between quantitative research and diachronic analysis. Specifically, some of the case studies and the questionnaire studies tend to follow positivism and deduce the reasons for differences in identity perceptions and succession behavior in terms of *post-hoc* results. In contrast, diachronic analysis considers everything as a process and needs to focus on its past, present, and future changes, thus emphasizing studies based on timelines and timing (Langley et al., 2013). Therefore, during succession, identity perception and expectation are not linear, but focus on changing priorities and are influenced by others in interaction (Lu et al., 2021), thus exhibiting iterative features, which can be better described through diachronic study.

Second, **comparative analysis** can be adopted to explore how culture, gender, and familiness influence succession. For example, in Asian (e.g., China, Korea) and European countries (e.g., Italy), the cultural background shows a strong sense of patriarchal control and collectivism, while the United States respects individualism, which leads to different family values and kinship, and result in diverse identity perception and succession behaviors (e.g., Sharma and Srinivas Rao, 2000; Porfírio et al., 2020). Similarly, compared with higher family control, the lower control of the business can create a distinct family-firm boundary, which may reduce role conflict and facilitate identity perceptions and succession behaviors (Cabrera-Suárez and Martín-Santana, 2012). Future research could also examine whether the perceptions and behaviors are specific to family firms by comparing family firm leaders with non-family firm leaders (Gagné et al., 2011). It would also be useful to explore differences in the understanding of gender across industries by comparing succession processes across industries and genders (Mussolino et al., 2019).

Third, text analysis and proxy variables should be introduced to measure identity perception. Most identity-based studies use case studies to analyze how an individual's identity perception influences their succession behaviors. For these propositions from case studies or theories, testing with quantitative data is the focus of future research. However, due to the specificity of the family business, questionnaire surveys cannot be popularized for data collection. Therefore, the text analysis method will be a substitution, as researchers can code identity perceptions and corporate culture and vision (Shepherd and Haynie, 2009) from the incumbent's and successor's speeches, annual reports, and homepage (Ahrens et al., 2019). Besides, new variables can be introduced as proxy variables for successors' self-concept in the succession, such as the frequency of successors attending company meetings and statements and the department in which the successors work when they first join the business.

Last, relevant theories and studies should be introduced to explain the mechanism of identity perception and behavior. For example, as suggested by Combs et al. (2020), the theory of **family science** is becoming one avenue to understanding behaviors in business families. As the incumbents aging, their authority weakens while their children's support obligations increase, signaling that the children's contribution to the family exceeds their consumption (Arkush, 1981). This transition may become obvious in a changing business environment. Then, the development of the successor's cognition also needs to be further explored, and **personality development theory** could be introduced in the future to explain how the birth order and personal experiences of the successor affect the development of his or her personality (Calabrò et al., 2018). In addition, current research has not explored the leader role identity of the successor sufficiently, so that cannot reveal how the successor should behave at each phase. The future study should draw on the ideas and findings related to **leadership** to refine the connotations of the leader role identity and the subsequent nurture and learning behaviors. Concerning the teaching and learning concepts, the present study can refer to **pedagogy-related theory** in leader identity construction. The construction of the successor's leader identity is not a simple process of resource transfer, but a more complex acquisition process of cognition, emotion, and skills, suggesting that family education and university education become significant situations for the successor socialization process. It remains unclear how to develop leadership skills for the next generations through appropriate education methods when we regard them as students. Education studies will also provide insights for leadership study, when there is a lack of kinship in education. Further, future research could introduce insights from evolutionary psychology (e.g., Sharma et al., 2020), and co-evolution theory (Lewin and Volberda, 1999), explain how an individual's identity perception changes over time, and analyze how the incumbent and successor influence each other. Last, future research could adopt set theory or configuration logic to explore how the combination of conditions such as intergenerational identity perceptions, intergenerational interactions, and the social context produce differentiated intentions and motivations, as well as identity behaviors and succession outcomes in family business succession (as fsQCA method adopted in Porfírio et al., 2020).

## 6. Conclusion

### 6.1. Summary

The identity perspective proposed in this article introduces the concepts of SIT and RIT, and combines several sociological and psychological theories to discuss the micro-foundations of family business succession, focusing the succession on identity perception and identity construction behaviors. Specifically, this review aims to answer the following questions: How does the identity concept explain antecedents and consequences of succession behaviors at different phases? And how does the identity concept motivate future research? To address these two research questions, this review codes and analyzes 99 articles, and composes a knowledge framework to summarize variables in the succession study, focusing on the proximal context of intergenerational interaction and the intergenerational feedback features. Then, this review proposes future research directions in terms of research topics, research methods, and perspectives within the existing knowledge framework, emphasizing the incumbent's experience, the connotation of leader identity, and the whole process. To this end, future research could consider methods such as textual analysis, longitudinal analysis, and comparative analysis, as well as theories such as family theory, personality development theory, leadership studies, and pedagogy-related theory.

### 6.2. Implications

This article makes four theoretical and practical contributions to succession research. First, by integrating the identity concept on the individual level into family business succession, this literature review regards succession as a process in which the incumbents and the successors confirm identities around phased objectives, clarifying the cognitive focus and behavioral outcomes of both the incumbent and the successor at different stages of succession. Further, the knowledge framework proposed in this study provides possible directions for future research. Second, this study provides diagnostic tools for participants in succession. Specifically, the knowledge framework motivated by identity perspective helps to recognize identity challenges for the incumbents, the successors, and the advisors, so that they can adjust identity perceptions and identity motivations in time to facilitate smooth succession. For example, on the one hand, at the beginning of the succession, the incumbent and the successor should first clarify the leader role expectation to set a shared vision for the succession. On the other hand, they are supposed to adjust their understanding of self-concept and avoid stereotypes to construct the successor's leader identity through practical decision-making. Third, this article emphasizes that succession is not a sequential, forward-flowing process where the successor replaces the incumbent (Magrelli et al., 2022), but an intersubjective and recursive process, where identity perceptions of the incumbent and the successor are interconnected and mutual feedback. Last, the identity perspective proposed in this article also helps to explore CEO succession in non-family business. Previous research on leadership succession in non-family firms has mainly considered external antecedents of involuntary succession, such as the board of directors, firm performance, human capital, and industry. However, recent studies have begun to consider mechanisms of voluntary succession, for example, the founder CEOs who have lower organizational identification with the firm usually take fast succession when faced with event shocks (Lee et al., 2020). The succession knowledge framework under the identity perspective thus provides a reference to inform leadership succession by exploring how psychological factors lead to voluntary succession.

Certainly, this review is not comprehensive in terms of identity perspectives. First, by focusing on individual identities, the framework is concentrated at the individual level, ignoring such variables as power structures in the firm, and political and economic factors. Second, this study focuses on two participants in the succession and regards managers and spouses as external variables that may influence identity perception. Last, this review is based on two types of identities: identity in the family and identity in the firm. In fact, the incumbents and the successors may assume more identities, such as alumni, political status, etc. Future research can also review the above aspects of family business succession.

## Data availability statement

The original contributions presented in the study are included in the article/Supplementary material, further inquiries can be directed to the corresponding author.

## Author contributions

WL: conceptualization, editing, and funding acquisition. YW: writing the original draft and review. LC: review and editing. All authors contributed to the article and approved the submitted version.
