# Supplementary material for: Identities of the incumbent and the successor in the family business succession: Review and prospects
Source: Front Psychol. 2023 Mar 17;14:1062829. doi: 10.3389/fpsyg.2023.1062829 (PMC10064008; doi:10.3389/fpsyg.2023.1062829)
Supplement: Supplementary file 1 [file Data_Sheet_1.docx]

Supplementary Material

**Appendix. An overview of 99 reviewed articles**

(Methods are abbreviated as follows: C=conceptual; QL=qualitative; QN=quantitative; M=mixed)

| **Authors** | **Method** | **Data** | **Key findings** | **Succession topic** | **Identity concept** | **Theoretical basis** |
| --- | --- | --- | --- | --- | --- | --- |
| Ahrens et al., 2015 | QN | Germany 787 | There exists gender preference for male family heirs in family business succession, which constrains the labor market and leads to male successors. | Successor choice, gender | Role expectation of the leader | Contest theory |
| Ahrens et al., 2018 | QN | Germany 804 | When the successor’s human capital is high, the incumbent’s involvement may lead to intergenerational conflict, failure in the successor’s leader identity construction, and outdated strategy, which will finally hinder firm performance. | Retention or retirement | Role conflict | Upper echelon theory |
| Ahrens et al., 2019 | QN | Germany 804 | Though family firm succession negatively influences firm performance, the successor's attributes like human capital, work experience in the family firm, and family member identity are significantly positively related to post-succession firm performance. | Successors' strategic decision | Self-perception of leader identity | Social exchange theory |
| Alterman et al., 2020 | C | / | The author outlines small business owners' 4 retirement decisions and provides multilevel antecedents that may shape SBOs' retirement decisions, including individual, relational, and business levels. | Transgenerational intention | Perception of leader identity | Self-expansion theory |
| Aygoren & Nordqvist, 2015 | QL | Sweden 1 | The research draws a framework on identity work that family members as the first generation adopt to form and manage family and business identities in the family business, which further leads to succession intention. | Transgenerational intention | Identification with the family and firm group | Identity theory |
| Barbera et al., 2015 | QL | America 8 | By exploring the consequence of the undergraduate family business program, the authors argue that whole-person learning can be a significant approach to educating the successor through the development of cognitive, emotional, and social skills. | Foster and development | Leader identity work | Family system theory |
| Barnes, 1988 | QL | America | Daughters and younger sons face serious role conflicts, which result in tensions among family members and can be dealt with by a third party’s helping in role recognition | Contradictory emotion and behavior, gender, birth order | Role conflict | Life cycle concept |
| Bertschi-Michel et al., 2020 | QL | Switzerland 5 | Advisor, as a trusted third party, can improve satisfaction in succession by identifying emotions. Specifically, advisors usually unearth incumbents’ and successors’ emotions to facilitate role adjustment between them. | Contradictory emotion and behavior | Role conflict | Emotion regulation theory |
| Bika et al., 2019 | QL | Scotland 1 | Successors' socialization process involves three layers: internal, interactive, and experiential. All three layers are interdependent, but the relevance and priority depend on the wider socioenvironmental context. | Foster and development | Leader identity work | Socialization theory |
| Björnberg & Nicholson, 2012 | M | UK 960 | Emotional ownership origins from the family climate, shaped by family climate, facilitated by a structured, organized, and open manner, which is necessary for the continuance of the family firm. | Takeover intention, commitment | Identification with the family firm | Social identity theory, emotional ownership |
| Bloemen-Bekx et al., 2021 | QL | Europe 1 | Business families use informal family governance mechanisms (including symbolic and interactive) to nurture successors' affective commitment to the family business before they join the family business. | Takeover intention, interaction | Identification with the family firm | Commitment theory |
| Byrne et al., 2019 | QL | France 4 | The Incumbent's and the successor's description of expected leadership contains a masculine nature, suggesting their role expectation of leadership identity and supporting the social construction nature of the successor role. | Successor choice, gender | Role expectation of the leader | Gender theory |
| Byrne et al., 2021 | QL | France 7 | Successors often 'do masculinity ' and 'do feminity' to pursue legitimacy, at the same time, sons and daughters adopt different strategies to garner support and build alliances. | Recognition tactics, gender | Leader identity work | Gender studies |
| Cabrera-Suárez et al., 2012 | QN | Spain 138 | Successors' affective commitment significantly influences success, while normative commitment takes indirect influence through affective commitment. | Successful succession, commitment | Identification with the family firm | Social identity theory |
| Cabrera-Suárez, 2005 | QL | Spain 7 | The success of succession depends on both successors’ commitment and leadership development, which are influenced by intergenerational relationships, successors’ motivations, as well as incumbents' support. | Foster and development, commitment | Leader identity work | leadership and commitment studies |
| Cadieux, 2007 | QL | Canada 5 | The author emphasizes that in stages 2 and 3, the incumbents are faced with a transition in their life when their roles as a leader are replaced and present a typology of incumbents' roles during these stages. | Foster and development | Leader identity work | / |
| Calabrò et al., 2018 | QN | Italy 843 | Motivated by SEW, the incumbents prefer primogeniture, which has a negative effect on firm performance. | Successor choice, successor's strategic decision | Role expectation of the leader, leader type | Socioemotional wealth |
| Canovi et al., 2022 | QL | Italy1 | Family cohesion fosters the entrepreneurial role identities of the successors through strategic actions, which could motivate them to pursue entrepreneurial opportunities. | Entrepreneurship, foster and development | Leader identity work | Role identity theory |
| Cater & Justis, 2009 | QL | America 6 | Six factors influence successors’ development: intergenerational relationship, knowledge, long-term orientation, cooperation, successor identity, and risk orientation. | Foster and development | Leader identity work | / |
| Chalus-Sauvannet et al., 2016 | QL | France, Canada 6 | The children who initially work outside the family firm experience career success and entrepreneurship, which motivate them to return and take over the family firm. | Takeover intention, career | Self-perception of leader identity | Succession planning studies |
| Chen et al., 2021 | QN | China 348 | Influenced by Confucianism, incumbents choose a family member or a guanxi-connected nonfamily member as the successor, who can acquire specialized assets through internal experience, which benefits firm performance. | Successor choice, performance | Identification with the family | National culture |
| Chlosta et al., 2012 | QN | Germany 461 | Parents' role model increases successors' entrepreneurship and self-employ intention, while successors' openness moderates this relationship. So that autonomy and creativity in the family business are necessary for succession intention. | Takeover intention, career | Self-perception of leader identity | Social learning theory |
| Chrisman et al., 1998 | QN | Canada 485 | The incumbents regard personal qualities, represented as “integrity” and “commitment to business” as the most important attributes of successors, instead of gender and birth order. Such factors become more important as the family firm grew. | Successor choice | Role expectation of the leader | / |
| Cooper et al., 2013 | C | / | The integrated nature of family and firm boundaries creates tension for family members, which leads to negative emotions and deviant behaviors in intergenerational succession. | Contradictory norms | Role conflict | Boundary identity theory |
| Curimbaba, Florence, 2002 | QL | Brazil 12 | Due to the gender stereotype, family and firm structure influence daughters’ possibility to become successors: invisible, professional, and anchor. | Successor choice | Role expectation of the leader | Gender studies |
| Dalpiaz et al., 2014 | QL | Italy 1 | The successors adopt narrative tools, including constructing a sense of family, family eulogizing and highlighting nonfamily endorsement to legitimate their leader identity and firm strategies. | Recognition | Leader identity work | Narrative studies |
| Dawson et al., 2015 | QN | Canada, Switzerland 199 | Successors' intentions to stay in the family firm are based on different commitments: identity and career interest improve affective commitment; family expectation result in normative commitment and financial wealth associated with continuance commitment. | Takeover intention, career, commitment | Identification with the family firm, Self-perception of leader identity | Social identity theory |
| DeNoble, 2007 | QL | US 7 | The incumbents regard social capital, represented as family relations and social relations, as well as human capital, represented as tacit, industry, and business knowledge as the most important attributes of successors. | Successor choice | Role expectation of the leader | / |
| Eckrich & Loughead, 1996 | QN | America 248 | Family business membership does influence successors' career development, as parents' overinvolvement results in successors' low commitment to career choice. | Takeover intention, career | Self-reception of leader identity | Vocational identity studies |
| Fang et al., 2023 | QN | China | The authors clarify the critical qualities of a competent successor with a hybrid model and illustrate effectiveness in an empirical case study. | Successor choice | Role expectation of the leader | Fuzzy theory |
| Feldmann et al., 2022 | QN | Europe 2897 | Gender identity influences the descendant's career choice, so daughters are more likely to be an employee vs successor vs entrepreneurs, while role expectations from the family member moderate this relationship. | Takeover intention, gender | Self-perception of leader identity | Culture and gender studies |
| Foster, 1995 | QL | America | The leadership development of the successor includes industry knowledge, business skills, influence skills, and self-awareness, so that the incumbent should create a developmental environment of challenge, recognition, and support. | Foster and development | Leader identity work | Leadership studies |
| Frederik & Riar, 2022 | QL | German, Austria 24 | The motivation of the leader role of the incumbent and successor induces venturing activities, as the incumbent fulfills entrepreneurial enthusiasm, and the successor gains acknowledgment. | Entrepreneurship, retirement, recognition | Leader role as motivation | Entrepreneurship studies |
| Gagnè et al., 2012 | QN | Canada 117 | Incumbents' goal adjustment ability, a psychological variable, including disengagement and reengagement, will influence their retirement planning. | Retention or retirement | relinquish leader role | Goal adjustment theory |
| Garcia et al., 2019 | C | / | Family relationships, including parental support and psychological control are important factors in shaping successors‘ engagement in the family firm, through self-efficacy and commitment. | Foster and development | Self-perception of leader identity | Social cognitive theory, |
| García-Álvarez et al., 2002 | QL | Spain 13 | The incumbent's value and business cognition determine the socialization patterns of the successor: the founder's homosocial reproduction model and the new leader development model. | Foster and development | Role expectation of the leader, leader identity work | Socialization theory |
| Gimenez-Jimenez et al., 2021 | QN | 33 countries 18576 | Affective commitment partially mediates the relationship between family business exposure and the successor's succession intentions. And this relationship is stronger for sons than for daughters, while birth order has no effect. | Takeover intention | Identification with the family firm | Family Science |
| Goldberg & Wooldridge, 1993 | M | America 254 | Effective succession happens when the successor is equipped with self-confidence and managerial autonomy, and gains recognition from the incumbent. | Autonomy | Self-preception of leader identity | / |
| Haberman & Danes, 2007 | QL | America 2 | Compared with women in the father-son business, women in the father-daughter business experience higher feelings of inclusion, resulting in lower conflict and integration among family members | Foster and development, gender | Leader identity work | Fundamental Relationship Orientation Theory (FIRO) |
| Handler, 1990 | QL | America 32 | Succession can be regarded as a mutual position adjustment process between the incumbent and successor. | Mutual acceptance | Leader identity work | / |
| Hauck & Prügl, 2015 | QN | Austria 81 | Family adaptability and a family member’s closeness to the firm are positively associated with perceiving the succession phase as an opportunity for innovation, and intergenerational authority and the history of family bonds have a negative effect. | Recognition, successor's strategic decision | Leader identity work | SEW |
| Huang et al., 2020 | M | China 260 | Successors' ability and willingness to take over the business influence incumbents' domination degree over the successors, and incumbents' family-work role integration degree moderates this relationship. | Control behavior | Role conflict | Intergenerational studies |
| Hytti et al., 2017 | QL | Finland 4 | Daughters construct and negotiate their leadership identity in their interactions with others by opposing, expanding, and making use of the gendered scripts available to them | Recognition tactics, gender | Leader identity work | Gender theory |
| Jaskiewicz et al. 2016 | QL | Germany 21 | The different ways that incumbents choose to manage conflicting family logic and commercial logic will lead to different succession decisions. | Transgenerational intention | Identification with the family and firm group | Institutional logic |
| Jaskiewicz et al., 2015 | QL | Germany 21 | Incumbents adopt entrepreneurial legacy, defined as the family's rhetorical reconstruction of past entrepreneurial achievements, to motivate successors to engage in strategic activity to foster transgenerational entrepreneurship. | Foster and development, entrepreneurship | Leader identity work | Imprinting theory |
| Kandade et al., 2021 | QL | India 24 | Successors develop a high-quality relationship with family and non-family stakeholders through strategies of respect, trust, early affiliation, mentoring, and mutual obligation. | Recognition tactic | Leader identity work | LMX theory |
| Klein, 2008 | C | / | While the identity confirmation strategy facilitates the succession process, family features, including family type, complexity, and life cycle, moderate this relationship. | Mutual acceptance | Identity confirmation | Family studies |
| Lam, 2011 | QL | China 12 | The incumbents' multi-roles lead to inconsistency between the expressed attitudes, perceptions, plans, and actual behaviors when they hand over the business to the next generation. | Contradictory emotion and behavior | Role conflict | Sensemaking |
| Lauto et al., 2020 | QN | Italy 148 | Compared with entrepreneurs, successors experience lower discretion, which leads to lower job satisfaction. | Discretion | Self-reception of leader identity | Self-determination theory |
| Le Breton-Miller & Miller, 2014 | C | / | Identity in the family formed in early times will influence the successor's leader identity. | Foster and development | Leader identity work | / |
| Lefebvre & Lefebvre, 2016 | QL | France 14 | Successors’ expectation of future leadership includes four types: the protector, reformer, opportunist, and rebel. These expectations might influence successors’ takeover intention. | Takeover intention | Role expectation of the leader | Role identity theory |
| Leotta et al., 2017 | QL | Italy 1 | Successors introduce management accounting practices to construct leader identity, through the integration of four dimensions of reality: facts, possibilities, values, and communication. | Recognition tactic | Leader identity work | Actor-reality construction |
| Li & Piezunka, 2020 | QL | China 7 | Successors’ and incumbents’ multi-roles in the family and business system will result in intergenerational conflict, which can be dealt with with the mother being the trusted third-party and setting a boundary between the family and the firm. | Clear boundary | Role conflict | Role identity theory |
| Lu et al., 2021 | QN | China 196 | A family CEO’s traditionality has a positive effect on the probability that a family member is chosen as successor. | Transgenerational intention | Identification with the family | Upper echelons theory |
| Mahto et al., 2019 | QN | US 945 | The level of overlap between family identity and business identity will influence family's transgenerational intention. The firm CEO's identification of the family moderates this relationship | Turnover intention, commitment | Identification with the family firm | Social identity theory |
| Mahto et al., 2019 | QN | America 945 | The level of overlap between family identity and business identity will influence the family’s transgenerational intention. Incumbents’ identification with the family moderates this relationship. | Transgenerational intention | Identification with the family | Psychological ownership theory |
| Mahto et al., 2021 | QN | US 168 | Incumbent's SEW influences the preference for a family successor and acceptance of the advisor role in the succession planning process. | Transgenerational intention | Identification with the family | SEW, Commitment theory |
| Mair & Rombach, 2020 | QL | Europe 30 | Successful succession requires the following skill from the successor: organizational and analytical skills, personal skills and attitudes, as well as knowledge-based skills. | Leadership skill | Role expectation of the leader | Work-family border theory |
| Martin et al., 2020 | QN | Spain 9146 | Incumbents adopt learning mechanisms to nurture successors' commitment to the family firm, which will influence their succession intention. | Takeover intention and behavior | Identification with the family firm | Dynamic capabilities |
| Martínez et al., 2020 | QL | Spain 4 | The expectation from the incumbents, successors, and stakeholders will support the successors' new leader work, which operates over time. | Foster and development | Expectations of the leader role | Work-motivation theory, attachment theory |
| Matthews et al., 1999 | C | / | Incumbents’ perception of successors’ competency influences their handover intention, and successors’ perception of leader identity decide their takeover intention and readiness. | Successor choice, takeover intention, mutual acceptance | Preception of leader identity | Cognition categorization theory |
| McAdam et al., 2021 | QL | Ireland 5 | During the succession process, the daughter co-constructs the leader identity with the father incumbent. Although daughters rely on certain intergenerational relations (preparation, endorsement, and osmotic credibility), they also need to develop independently to heighten their own visibility and credibility. | Recognition, gender | Leader identity work | Identity work and legitimacy |
| McMullen & Warnick, 2015 | C | / | The incumbents promote affective commitment in successors by supporting their psychological needs for competence, autonomy, and relatedness within the family business. | Takeover intention, commitment | Identification with the family firm | Self-determination theory |
| Meroño-Cerdán, 2022 | QN | Spain 177 | Daughters' features are preferred to cope with poor business performance and unsatisfactory family relationships. however, this unplanned successor may be a trap rather than an opportunity. | Successor choice, gender | Role expectation of the leader | Theory of planned behavior |
| Miller, 2014 | QN | America 100 | A shared vision for the family business predicts the leadership effectiveness and work engagement of next-generation, family climate (including open communication and intergenerational authority) influence the shared vision. | Foster and development | Leader identity work | Leadership studies |
| Milton, 2008 | C | / | Incumbents and successors face identity challenges in phase 3, so that they are supposed to confirm their identities, that is, incumbents exit leader identity, and successors complete leader identity with high status. | Mutual acceptance | Identity confirmation | Role identity theory |
| Mitchell et al., 2009 | C | / | Successors' agency in entrepreneurship, an individual dimension of managerial discretion, is constrained by individual traits, and this lack of agency is exacerbated by family firm-level factors, including wealth preservation and incumbent involvement. | Discretion | Self-reception of leader identity | Human agency lens |
| Murphy & Lambrechts, 2015 | QL | Ireland 6 | Successors' involvement in the family business in early times will influence their career choice, as the experience to help the family firm in childhood surpasses their career interests. | Takeover intention, career | Self-perception of leader identity | Vocational identity |
| Murphy et al., 2019 | QL | Ireland 6 | Key events in the early lives of successors improve their sense of belonging and identity, which become the heart of their socioemotional wealth and influence their succession intentions. | Takeover intention | Identification with the family firm | Life course theory |
| Mussolino & Calabrò, 2014 | C | / | Incumbent's paternalistic leadership influences the attitudes, subjective norms, and perceived behavioral control of successors in family firms, which further harms the successor's perception of the succession process. | Foster and development, control | Self-preception of leader identity | Theory of planned behavior |
| Mussolino et al., 2019 | QL | Italy 4 | Female successors' construction of self-concept depends on the incumbent's leadership style and whether their succession is accepted by employees. | Recognition tactic, gender | Leader identity work | Self-positioning theory |
| Overbeke et al., 2013 | QL | America 20 | Daughters may overlook their succession possibility, this may result from automatically activated gender norms, which impedes daughters' ascendancy. | Takeover intention, gender | Self-preception of leader identity | Theory of planned behavior, gender Role Theory |
| Overbeke et al., 2015 | QN | America 95 | The shared expectation of the daughter's leadership identity between the incumbent and successor will influence the daughter's possibility to be transformed into a successor, as gender bias and the daughter's efficacy influence role expectation. | Successor choice, gender | Perception of the daughter | Social cognitive theory, intentional change theory |
| Porfírio et al., 2019 | QN | Portugal, Greece 128 | Different configurations of the successor's individual characteristics, succession motivation, firm characteristics, and succession plan influence the successor's preparation for succession. | Succession preparation | Self-perception of leader identity | Expectation states theory |
| Porfírio et al., 2020 | QN | 6 countries 383 | The interplay between the personal characteristics of the successor, the organizational characteristics of the family business, and the context leads to different patterns of succession motivation. | Succession intention | Self-perception of leader identity | Entrepreneurship theory |
| Pruthi & Tasavori, 2022 | QL | UK 11 | The successors are heterogeneous in firm growth strategies, which depend on their succession context and use of social ties. | Successors' strategic decision | Self-perception of leader identity | Network theory |
| Querbach et al., 2020 | QN | Switzerland 205 | Sen-gens' retention negatively affects product innovation, and this negative effect is strengthened when sen-gen involvement in the successor selection process, and family ties. | Retention or retirement | relinquish leader role | Stakeholder salience theory |
| Radu-Lefebvre & Randerson, 2020 | QL | France 20 | Role conflicts will lead to the paradox of control and autonomy for successors, and will further trigger emotional ambivalence toward family members. Thus, successors may adopt defensive or confrontational strategies to pursue legitimacy. | Contradictory emotion and behavior | Role conflict | Paradox theory |
| Romaní et al., 2022 | QN | Latin America, 16185 | The affective and normative commitment and the parental role model determine the next generations' intention to be a successor. | Takeover intention | Identification with the family firm | Theory of planned behavior |
| Salvato & Corbetta, 2013 | QL | Italy 4 | Advisors play a transitional role in succession to co-construct the successor's leader identity through formal training and consulting on challenges faced by the successors. | Foster and development, recognition | Leader identity work | Leadership Construction Process |
| Samei & Feyzbakhsh, 2015 | QL | Iran 4 | Incumbent competencies required for nurturing are open-mindedness, risk-taking, patience, explicitness, motivation, trustworthiness, communicative skills, and value orientation. | Foster and development | Role expectation of the leader | Succession studies |
| Sardeshmukh & Corbett, 2011 | QN | America 119 | Family firm-specific human capital provides successors with entrepreneurial self-efficacy, a cognitive skill that is important for strategic opportunity perception and entrepreneurial venturing. | Opportunity perception | Self-perception of leader identity | Cognitive theory |
| Schell et al., 2020 | QL | Germany 12 | The next-generation sents positive signals such as hard-to-fake signals and costly signals to be selected as successors, or negative signals to be excluded from the succession. And this signaling process evolves over the selection process lifespan. | Takeover intention and behavior | Leader identity work | Signaling theory |
| Schenkel et al., 2016 | QN | Korea 400 | Successors' birth order influences the distribution of control through ownership, leadership (i.e., CEO), and the incorporation of outside board participation and governance | Successor's strategic decision, birth order | leader type | Role identity theory |
| Schröder & Schmitt, 2013 | QN | German 152 | Incumbents' support and jun-gens' perceived entrepreneurial competence predicted career interest in succession, whereas incumbents' control related to the family obligation to succession. | Takeover intention, career | Leader role as motivation, identification with the family group | Self-determination theory |
| Schroeder et al., 2011 | QN | Germany 106 | Personality traits, gender, identification with the family firm, and parental behaviors may influence successors' career choice intention. | Takeover intention, career | Identification with the family firm, Self-perception of leader identity | Theory of planned behavior |
| Shanine et al., 2022 | M | US 119+24 | Incumbents' parenting style affects successors’ psychological functioning, which impacts employees’ citizenship and counterproductive behaviors. | Foster and development, recognition | Self-perception of leader identity | Parental control theory |
| Sharma & Irving, 2005 | QL | Canada 4 | Different types of commitment take no difference in successors' takeover intention, however, affective commitment may result in better firm performance. | Takeover intention, commitment | Identification with the family firm | Social identity theory |
| Sharma & Srinivas, 2000 | QL | India 43 | Incumbents rate integrity and commitment as the two most important attributes of a successor. However, Indian owners rate blood and family relationships higher. Canadian owners rate interpersonal skills, past performance, and experience higher. | Successor choice | Role expectation of the leader | / |
| Sharma et al.,2001 | C | / | The incumbent's and successor's acceptance of self and family member's roles will influence the satisfaction with the succession process | Mutual acceptance | Identity confirmation | Stakeholder theory |
| Stavrou & Swiercz, 1998 | QN | America 130 | Successors' intention to join the family firm depends on personal elements including needs, goals, and abilities, and family elements including intergenerational relationships and values. | Takeover intention, career | Self-reception of leader identity | / |
| Torres et al., 2023 | QN | Global 33390 | Institutional and in-group collectivism practices increase next-generation's engagement | Foster and development | Self-perception of leader identity | Theory of planned behavior, institutional theory |
| Venter & Boshoff, 2006 | QN | South Africa 332 | Incumbent and successor’s acceptance of mutual identities, harmonious intergenerational relationships, and succession consensus will lead to successful succession. | Mutual acceptance | Identity confirmation | Identity theory |
| Vera & Dean, 2005 | QL | America 10 | Due to the gender stereotype, daughters usually meet challenges when constructing leader identity, which becomes serious when the incumbent is the mother, as the daughter is compared with her mother’s managerial style. | Recognition | Leader identity work | Gender and role identity studies |
| Wang & Zhang, 2022 | M | China 18+131 | Successors' intrapreneurial intention is related to perceived family relational conflict, mediated by family expectation and family relational self-efficacy. | Entrepreneurship | Self-perception of leader identity | Social cognitive theory |
| Wielsma & Brunninge, 2019 | QL | Holland 1 | Family business identity influences the identity of family members in early times, the founding members develop the business identity based on their values; in the latter stages, the identity of the business influences the family and its members. | Takeover intention | Identification with the family firm | Identity theory |
| Xian et al., 2021 | QL | China 20 | Chinese daughters adopt three approaches to construct identity in the family business: comply with traditional gender expectations, act as the second leader, and challenge to become an independent leaders. | Takeover intention, gender | Self-perception of leader identity | Role identity theory |
| Yoo et al., 2014 | QN | Korea 400 | Non-first son successor usually leads to better firm performance than primogeniture, as non-first sons are much more open to outside factors. | Successor's strategic decision, birth order | Leader type | Boundary identity theory |
| Zhu & Zhou, 2022 | M | China 122 | Incumbent's support increases successors' takeover intention, through self-efficacy and perceived person-job fit. | Takeover intention, self-efficacy, career | Self-perception of leader identity | Attachment theory |
